# Treatment of Urethral Strictures in Transmasculine Patients

**DOI:** 10.3390/jcm10173912

**Published:** 2021-08-30

**Authors:** Mieke Waterschoot, Wietse Claeys, Piet Hoebeke, Wesley Verla, Marjan Waterloos, Michel Wirtz, Marlon Buncamper, Nicolaas Lumen

**Affiliations:** 1 Department of Urology, Ghent University Hospital, 9000 Gent, Belgium; Wietse.Claeys@uzgent.be (W.C.); Piet.Hoebeke@uzgent.be (P.H.); Wesley.Verla@uzgent.be (W.V.); marjan.waterloos@hotmail.com (M.W.); michelwirtz5@hotmail.com (M.W.); nicolaas.lumen@uzgent.be (N.L.); 2 Department of Plastic and Reconstructive Surgery, Ghent University Hospital, 9000 Gent, Belgium; Marlon.buncamper@uzgent.be

**Keywords:** transmen, transgender, urethral stricture, sex reassignment surgery

## Abstract

Background: Urethral strictures are a common complication after genital gender-affirming surgery (GGAS) in transmasculine patients. Studies that specifically focus on the management of urethral strictures are scarce. The aim of this systematic review is to collect all available evidence on the management of urethral strictures in transmasculine patients who underwent urethral lengthening. Methods: We performed a systematic review of the management of urethral strictures in transmasculine patients after phalloplasty or metoidioplasty (PROSPERO, CRD42021215811) with literature from PubMed, Embase, Web of Science and Cochrane. Preferred Reporting Items for Systematic reviews and Meta-Analysis-(PRISMA) guidelines were followed, and risk of bias was assessed for every individual study using the 5-criterion quality appraisal checklist. Results: Eight case series were included with a total of 179 transmasculine patients. Only one study discussed the management of urethral strictures after metoidioplasty. Urethral strictures were most often seen at the anastomosis between the fixed and pendulous urethra. For each stricture location, different techniques have been reported. All studies were at a high risk of bias. The current evidence is insufficient to favor one technique over another. Conclusions: Different techniques have been described for the different clinical scenarios of urethral stricture disease after GGAS. In the absence of comparative studies, however, it is impossible to advocate for one technique over another. This calls for additional research, ideally well-designed prospective randomized controlled trials (RCTs), focusing on both surgical and functional outcome parameters.

## 1. Introduction

Genital gender-affirming surgery (GGAS) can be part of the transition process in transgender patients. The two standard options for transmasculine patients are phalloplasty and metoidioplasty. In metoidioplasty, the hormonally enlarged clitoris is converted to a (small) neophallus [1], while phalloplasty comprises the construction of a neophallus with different types of flaps [2]. Most of the patients undergoing this type of surgery also undergo a urethral lengthening procedure, as they have a strong desire to void in a standing position [3]. This, in turn, brings along the risk of complications at the neo-urethra, such as fistulas or urethral stricture formation [4]. 

Urethral strictures pose a specific challenge to the reconstructive urologist, and studies that focus on the management of urethral strictures in transmasculine patients are scarce. The management of urethral stricture disease in cisgender men includes endoluminal treatment (dilatation, internal urethrotomy) and surgical reconstruction of the urethra (urethroplasty), using different techniques according to patient and stricture characteristics [5]. This management cannot simply be extrapolated to transmasculine patients, due to several differences such as anatomy, paucity of local tissue, precarious vascularization and stricture etiology. 

The European Association of Urology guidelines on urethral strictures include a chapter dedicated to disease management in transmasculine patients [6]. However, the recommendations put forward are not underpinned by a systematic review of the literature. Therefore, the aim of this systematic review is to collect all available evidence on the management of urethral strictures in transmasculine patients who underwent urethral lengthening during metoidioplasty or phalloplasty. To the best of our knowledge, no such review has been performed so far.

## 2. Materials and Methods

### 2.1. Search Strategy, Selection of Studies, and Data Extraction

The EMBASE, PubMed, Web of Science and Cochrane database were searched for original articles written in English, French, German or Dutch. No starting date was set, and databases were searched until April 2021.

For this systematic review, the authors followed the Preferred Reporting Items for Systematic reviews and Meta-Analysis (PRISMA) 2020 statement [7]. The a priori study protocol was registered on PROSPERO, which contains a detailed overview of the entire search string (CRD42021215811). 

After conducting the literature search and removing duplicates, records were screened for eligibility by two authors working independently (MiWa and WiCl). Any discrepancies between them were discussed until a consensus was found. Any remaining conflicts were reviewed by a third author (WeVe), acting as a referee. After that, full texts were retrieved for the selected records and an identical procedure was deployed to verify the eligibility of studies based on their full text. The same 2 authors independently performed the data extraction and risk of bias (RoB) assessment. Any conflicts or queries were reviewed by a third author (WeVe).

Extracted information from all eligible records included general study information, sample size, surgical technique of GGAS and characteristics (type of flap if applicable, type of urethral lengthening, flap related complications), stricture characteristics (diagnosis and evaluation method, time-to-onset, count, length, localization), the presence of fistulas and their characteristics (time-to-onset, count, localization), previous procedures in urethral stricture management, type of intervention used in study, hospital stay, catheter stay, postoperative complications, follow-up time, stricture recurrence and characteristics (diagnosis and evaluation method, time-to-onset), need for definitive urinary diversion and patient satisfaction (as defined by the investigator). For the purpose of standardization between post-metoidioplasty and post-phalloplasty strictures, we named strictures based on their anatomical location. Strictures of the pendulous part of the urethra are strictures located at the phallic part of urethra post-phalloplasty and strictures in the extended urethra (e.g., longitudinal island flap) post-metoidioplasty. Strictures of the pars fixa are strictures located on the side of the tubularization of the inner surface of the labia minora, both post-phalloplasty and post-metoidioplasty.

### 2.2. Types of Study Designs Included

Randomized controlled trials, non-randomized comparative studies and single-arm studies (case series) were included, providing a minimum mean/median follow-up of 3 months. Case reports, narrative or systematic reviews, abstracts only, conference papers and letters to editor were excluded.

### 2.3. Types of Participants Included

Adult (≥18 years) transmasculine patients that underwent GGAS by either metoidioplasty or phalloplasty and presented with a urethral stricture for which intervention was carried out were included. Series including a mixed group of cisgender males and transmasculine patients, and not reporting separate outcomes for these groups were excluded.

### 2.4. Types of Interventions Included

Any of the following interventions were eligible for inclusion: dilatation (any form), Otis urethrotomy, direct vision internal urethrotomy (DVIU), meatotomy, meatoplasty, Heineke-Mikulicz stricturoplasty (HMS), graft augmented urethroplasty (GAU) (any type of graft allowed), flap augmented urethroplasty (FAU) (any type of local flap allowed), anastomotic repair (AR) with or without graft augmentation, staged urethroplasty with or without graft augmentation and definitive perineal urethrostomy (PU).

### 2.5. Types of Outcome Measures Included

The primary outcome was urethral patency rate at 3 months after stricture management. Secondary outcomes included postoperative complications within 3 months and patient satisfaction at 3 months postoperative (as defined by the investigator). 

### 2.6. Assessment of Risk of Bias

As all of the included studies were case series, the 5-criterion quality appraisal checklist for case series assessment was used. This tool consists of 5 questions:

1. Was there an a priori protocol?
2. Was the total population included or were study participants selected consecutively?
3. Was outcome data complete for all participants and any missing data adequately explained/unlikely to be related to the outcome?
4. Were all pre-specified outcomes of interest and expected outcomes reported?
5. Were primary benefit and harm outcomes appropriately measured?

If ‘no’, the study is at ‘high’ risk of bias. If the answer to all 5 questions was ‘yes’, then the study was at ‘low’ risk of bias. This is a pragmatic approach informed by the methodological literature [8,9,10]. 

### 2.7. Data Analysis

As no RCT was identified, a meta-analysis was not appropriate. A narrative synthesis was used instead. A subgroup analysis was planned for type of GGAS (metoidioplasty versus phalloplasty), site of urethral stricture (meatal, pars pendulans, anastomosis pars fixa-pars pendulans, pars fixa, anastomosis native urethra-pars fixa, native urethra) and primary versus recurrent stricture. However, this was not possible due to the low level of evidence of studies, and therefore a narrative review of outcomes according to stricture location and type of urethroplasty was performed. 

## 3. Results

### 3.1. Quantity of Evidence Identified

**Figure 1:**
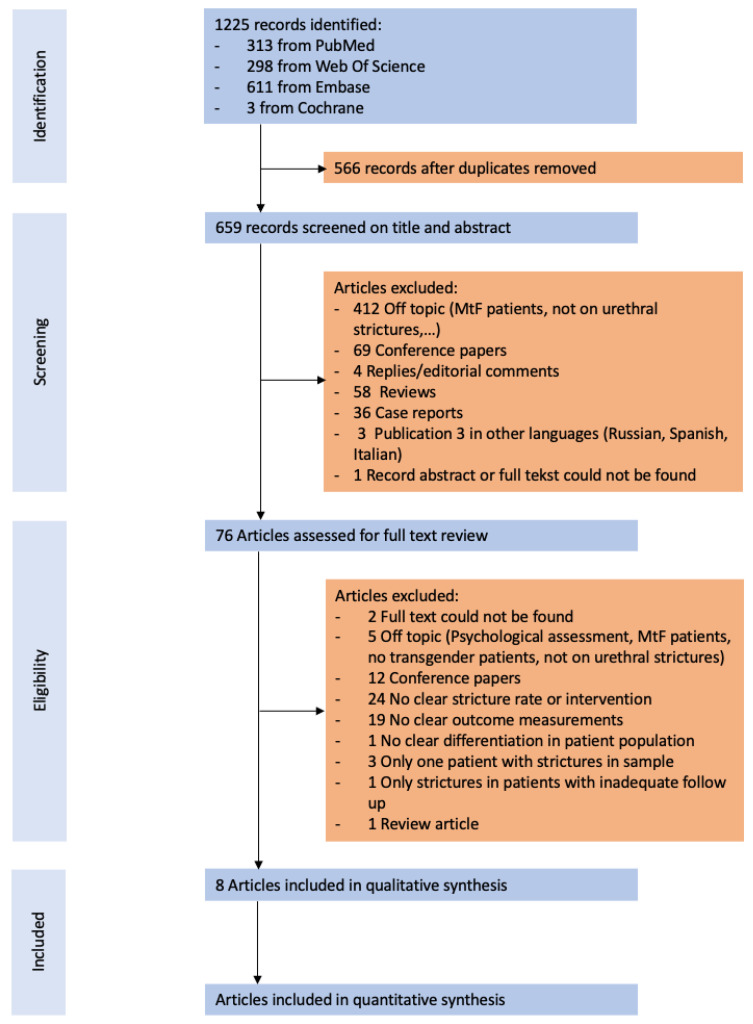
PRISMA flow diagram for study selection.

After removal of duplicates, 659 records remained, of which 76 were selected for full text review. For two of the eligible records, a full text could not be retrieved. Figure 1 shows a complete description of identification, screening and eligibility assessment of the identified records. In total, eight publications met the predefined inclusion criteria [11,12,13,14,15,16,17,18], with a total of 179 eligible transmasculine patients. Table 1 shows the qualitative summary of the extracted information in the sample population. Of these eight publications, two showed overlapping data [12,15]. The overlapping data concerned anastomotic repair (AR) urethroplasty, one of the various types of procedures used in Lumen et al. [12], and the sole type of studied procedure in Verla et al. [15]. For this reason, all EPA urethroplasties performed in Lumen et al. [12] were excluded from further assessment.

### 3.2. Characteristics of Included Studies

All included studies were case series. Mean/median follow-up of included studies ranged from 9 to 51 months (Table 1). 

Only one study discussed the management for urethral strictures after metoidioplasty (13 patients) [16]. All others described stricture management after phalloplasty (165 patients), except for one study in which one metoidioplasty case was included [17]. Overall, this accounts for the management of 224 urethral strictures in transmasculine patients. Both in phalloplasty and metoidioplasty, the fixed part of the urethra was reconstructed by tubularization of the vestibular mucosa between the native urethral meatus and the tip of the clitoris. The pendulous part of the urethra was reconstructed using a preputial skin flap or a labium minus flap in metoidioplasty cases. In the included studies, the phalloplasty’s were all performed in one stage. This means the combination of performing a vaginectomy, creating a neo-scrotum, constructing the fixed part of the urethra and making the connection between the fixed part and the pendulous part of the phallus. A radial forearm free flap (RFFF), anterolateral thigh flap (ALT), superficial circumflex iliac artery perforator flap (SCIAP) or an abdominal flap (AF) were used in phalloplasty cases, of which RFFF and ALT were the most common approaches (Table 1). 

### 3.3. Patterns of Strictures after GGAS

Time to stricture onset after metoidioplasty ranged from 12 to 17 months [16]. These strictures were mostly located at the anastomosis between the native and fixed urethra (33%), or the anastomosis between the fixed and pendulous urethra (33%). Stricture length after metoidioplasties was not reported. After phalloplasty, time to stricture onset ranged from 6 to 36 months [11,12,15]. In most studies, the anastomosis between the fixed part and pendulous part was the dominant stricture area. No strictures in the native urethra were reported. 

Five studies reported on the presence of concomitant fistulas [11,14,15,16,17], which ranged from 0–50%. Six studies reported on previous stricture related interventions [11,14,15,16,17,18]. Two of these [14,16] reported on patients without any prior interventions, although Wilson et al. [14] remained unclear on the use of previous endoscopic procedures. In the study of Verla et al. [15], 25% (11/44) of patients had previous endoscopic procedures and 39% (17/44) had previous urethroplasty. Pariser et al. [17] reported that all nine cases (100%) underwent at least one DVIU prior to urethroplasty. Of these, 22% (2/9) also underwent previous urethroplasty. In the study of Lumen et al. [11], 36% (8/22) of patients underwent previous urethroplasty. Dabernig et al. [18] reported that all their six cases (100%) underwent previous endoscopic or open treatment but remained unclear on the types and numbers. 

### 3.4. Patency Rates of Different Techniques

#### 3.4.1. Minimally Invasive Procedures

In three studies, minimally invasive procedures (Otis, DVIU, meatotomy and HMS) were used as stricture management [11,12,14] after phalloplasty. In the series exclusively reporting on DVIU (n = 22) [11], a first DVIU yielded a patency rate of 46%, whereas three or more DVIUs in the same patient were never successful and could not yield any valuable patency. They also calculated a mean time to stricture recurrence of 3 (range 2–3) months and 9 (range 1–54) months after one DVIU for strictures in the pendulous urethra and anastomotic strictures between the pendulous and fixed urethra, respectively. In Lumen et al. [12], 8/118 (6.8%) of strictures were treated with meatotomy and 19/118 (16%) with HMS with a patency rate of 75% and 58% respectively. Wilson et al. [14] managed 1/4 (25%) strictures with HMS without recurrence. The treatment for one meatal stenosis was not reported. 

Two out of twelve (17%) post-metoidioplasty strictures were managed with meatotomy and 3/12 (25%) with HMS, yielding patency rates of 50% and 67%, respectively [16].

#### 3.4.2. Urethroplasty

##### Graft Augmented Urethroplasty (GAU)

Four studies reported on GAU, and all of them in phalloplasty patients [12,13,14,17]. Schardein et al. [13] treated all their nine strictures with a double faced buccal mucosa graft (BMG) in a dorsal inlay and a ventral onlay approach, reaching a 75% urethral patency rate (only eight included cases, one case had no information on follow-up). Wilson et al. [14] reported on the use of dorsal inlay BMG urethroplasty in 2/4 (50%) strictures without any recurrence. All of these were reinforced with a local fasciocutaneous flap to support blood supply. Pariser et al. [17] treated 8/9 (88.9%) strictures with ventral onlay BMG, and the other case (11.1%) was ventral BMG augmented anastomotic repair. This resulted in a urethral patency rate of 56%. They reported a mean time to stricture recurrence of 7 (range 1–21) months after their augmented BMG repair. Lumen et al. [12] used graft urethroplasty in 2/118 (1.7%) strictures with a 50% urethral patency. The type of graft was not specified. 

##### Pedicled and Free Flaps

Local and distal flap reconstructions were used in three papers [12,16,18]. A labium minus flap was used in 1 out of 12 (8.3%) post-metoidioplasty cases without stricture recurrence [16]. Lumen et al. [12] described the use of a pedicled flap urethroplasty in 10/118 (8.5%) of cases (respectively seven and three neophallic skin and neoscrotal skin flaps). The overall patency rate of this technique was 60%. Dabernig et al. [18] performed a complete reconstruction of the pendulous urethra for multifocal strictures using radial forearm flaps in all their cases (six patients) with a urethral patency of 67%.

##### Anastomotic Repairs (AR)

Verla et al. [15] described the use of AR in all their reported strictures (44 cases), all located at the anastomosis between the fixed and pendulous part of the urethra. They reached a urethral patency rate of 57%. 

##### Staged Repairs

Staged repairs were discussed in two studies [12,16]. After metoidioplasty, this technique was used in 6/12 (50%) cases, yielding a patency rate of 33% [16]. After phalloplasty, staged Johanson urethroplasty was used in 33/118 (28%) cases with a 70% urethral patency [12]. Temporary perineal urethrostomy before urethral reconstruction was performed in 21/118 (18%) cases with a reported urethral patency rate of 38%. Another 10/118 (8.5%) cases underwent a first stage of a planned staged Johanson urethroplasty, or had a current perineal urethrostomy and were awaiting further treatment. One of the patients with a temporary perineal urethrostomy opted to maintain this state to avoid any further complications.

**Figure 2:**
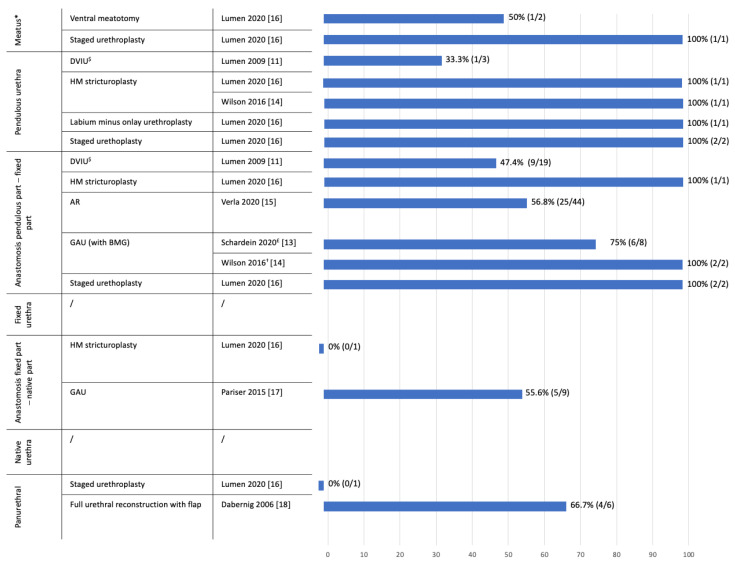
**Percentage of urethral patency per type of urethral repair stratified by location.** Lumen et al. [12] was not taken into account in this figure, since no clear patency rates per location and per type of urethroplasty could be withdrawn from this study. * Wilson 2016, no information on treatment and follow-up of one meatal stricture, ^$^ only first DVIU included, ^£^ (1 patient no information on follow-up, not included in recurrence rate), ^†^ all with fasciocutaneous flap reinforcement. DVIU (Direct Vision Internal Urethrotomy), HM (Heineke Miculicz), AR (Anastomotic Repair), GAU (Graft Augmented Urethroplasty), BMG (Buccal Mucosal Graft).

Figure 2 depicts the urethral patency rates of all different types of stricture repair, stratified by location based on the overall follow-up time of each study. Lumen et al. [12] could not be added to this figure, since they did not report on separate patency rates per stricture localization. 

### 3.5. Postoperative Complications

Four studies reported on postoperative complications [15,16,17,18]. Lumen et al. [16] reported that none of the patients experienced a grade 3 Clavien-Dindo (CD) complication after their various techniques for metoidioplasty. They did not report on the number of grade I and II complications. Verla et al. [15] reported that 5/44 (11%) patients experienced a CD grade I complication, 6/44 (14%) CD grade II and 1/44 (2.3%) a CD grade III complication after AR. The grade I and II complications involved urinary tract infections (UTI’s), wound infections, fistulas, hematomas and retention. The CD class III case involved insertion of a suprapubic catheter for urinary retention. Pariser et al. [17] described a CD grade II complication in 1/9 (11%) after their graft urethroplasties. This involved a mild rhabdomyolysis. Dabernig et al. [18] reported having no postoperative complications after their full free flap reconstructions (0/6). 

### 3.6. PROMs and Satisfaction

Two studies reported on patient reported outcome (PROM) use [13,18]. Schardein et al. [13] stated that 7/8 (88%) patients (only those with available data included) were able to void while standing, and reported a mean postoperative International Prostate Symptom Score (IPSS) of 3.1 (range 0–11) and an IPSS-QoL of 0.9 (range 0–3). However, they did not provide any preoperative data. On a global response assessment question (GRA), 6/8 (75%) patients reported a marked improvement, 1/8 (13%) a moderate improvement and 1/8 (13%) a slight improvement. Dabernig et al. [18] stated that all patients (six cases) reported an improvement in their mental well-being, and stated that they would undergo the procedure again if they would have to. However, these parameters were not assessed preoperatively. 

### 3.7. Risk of Bias Assessment

**Figure 3:**
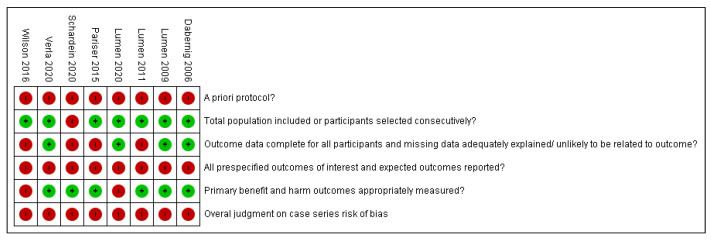
Risk of bias assessment per included study, using the 5-criterion quality appraisal checklist. Green: Low risk of bias, Red: High risk of bias.

Risk of bias was high in all included studies. Figure 3 depict the detailed risk of bias assessment per study.

## 4. Discussion

### 4.1. Study Findings

In this systematic review, the majority of patients were treated for strictures at different locations after phalloplasty. Only 13 patients were treated for post-metoidioplasty stricture. This discrepancy can be explained by the fact that transmasculine patients are more likely to choose a phalloplasty rather than a metoidioplasty, resulting in a higher absolute number of documented phalloplasty related stricture cases. Another reason might be the fact that urethral complications (strictures/fistulas) are less likely after a metoidioplasty than after a phalloplasty, given the less elaborated reconstruction and the less invasive type of tissue transfer. However, Waterschoot et al. reported urethral complications after metoidioplasty in 19%, whereas after phalloplasty this is in the same range [19].

For a meatal stenosis after metoidioplasty, Lumen et al. [16] reported a 1/3 (33%) urethral patency rate after ventral meatotomy and 1/1 (100%) after staged urethroplasty. The low patency rate after meatotomy could be explained by the intrinsic diminishment in the vascularization of the mobilized skin and clitoris, to create the fixed and pendulous urethra after metoidioplasty, while the tissue is less inflammatory and possibly better vascularized during the second stage of a staged urethroplasty. This is purely hypothetical as this is not described in the literature.

For meatal stenosis repair after phalloplasty, Lumen et al. [12] treated eight meatal strictures with a meatotomy yielding a patency rate of 75%. The other 10 were treated with a pedicled flap repair (five cases) or a staged repair (five cases), but separate outcomes were not reported. Due to these small patient numbers, no conclusions can be drawn on the preferred technique in this type of patient. However, different local factors can influence the choice of the technique that is performed. For example, if the patient is satisfied with a hypospade meatus, a meatotomy can be a straightforward and relatively simple solution. Otherwise, more complex options, such as a local flap urethroplasty or a staged repair might be necessary. 

When considering strictures at the pendulous urethra after metoidioplasty, three different surgical techniques were reported. Lumen et al. [16] performed a HMS, staged urethroplasty and labium minus flap urethroplasty in respectively one, one and two patients with a 100% patency rate [16]. So, it appears that strictures at the pendulous urethra after metoidioplasty are treatable, although larger studies are needed to confirm these results and to better understand the outcomes of each type of surgery. Here, again, multiple techniques for stricture treatment are possible depending on several patient and stricture characteristics.

Regarding pendulous strictures after phalloplasty, DVIU (11) has been attempted in only three cases with recurrence in two patients. In cisgender men, DVIU is not recommended for penile strictures, and based on the very limited experience, DVIU seems to have a limited role in the treatment of pendulous strictures in transmasculine individuals [6]. 

Lumen et al. [12] reported 28 strictures at the pendulous urethra. These were most commonly treated with a staged urethroplasty or a temporary perineal urethrostomy. However, separate outcome data per stricture location could not be obtained from this study. Another option is an RFFF as a complete urethral substitute, as described by Dabernig (REF invoegen). As this is an extensive and complex procedure with (additional) visible scarring at the forearm, this technique should be reserved in case (almost) the entire pendulous urethra is strictured and scarred. However, given the low patient numbers and high risk of bias, no definitive recommendations can be made on the ideal treatment of strictures at the pendulous urethra. 

Strictures at the anastomosis between the fixed and pendulous urethra were most frequently reported (125/224 strictures) (Table 2). The commonly used techniques in this anatomic region were AR, GAU with BMG, DVIU and HMS in respectively 44, 10, 19 and 16 strictures [11,12,13,14,15,16]. A patency rate of 75% (6/8 cases) and 100% (2/2 cases) was seen after GAU with BMG [13,14]. The success rates after DVIU, AR and GAU are respectively 37% (7/19) and 57% (25/44) at this location [11,15]. In cisgender males, DVIU is a potential first-line treatment for short and primary bulbar strictures, with a patency rate ranging between 26% and 77% being found after a single session [20]. Furthermore, Lumen et al. [11] showed that the shorter the time interval between phalloplasty and DVIU, the higher the risk of urethral stricture recurrence. Therefore, DVIU could be a potential first-line option as well for short (<3 cm) and primary anastomotic strictures that occur in the long run after phalloplasty [11].

Although AR is often associated with an excellent patency rate in cisgender men (93–97%), these favorable outcomes were not reached in transmasculine patients. These differences in success rates between cisgender and transgender patients could be explained by different facts. In general, vascularization is compromised at the proximal and distal end of the reconstructed skin urethra, due to the anatomy of free and pedicled skin flaps [21]. The new connection is one between the mucosal tissue and skin, which could explain the formation of more scar tissue after healing. Furthermore, safely mobilizing the neo-urethra without further compromising its vascularization is hardly possible, which makes it very difficult to create a tension free anastomosis. This is in contrast to cisgender men, in which a pure mucosal anastomosis is feasible, and mobilization of the urethra is much easier without compromising the vascularization, due to the natural curve it contains. Thus, as suggested by Verla et al. [15], probably only very short anastomotic strictures (<2 cm) with a peri-operatively assessed and good vascularization might be treated successfully with this technique, provided that a tension free anastomosis can be made. Based on the data of Lumen et al. [16] and Schardein et al. [13], a BMG or two stage urethroplasty might be a valuable alternative when there is any doubt on the quality of the tissue or tension of the anastomosis, but comparative studies are needed to confirm these results. Despite the lack of native supportive tissue (corpus spongiosum) for fixating a local flap or graft, Schardein et al. [13] showed a 78% (7/9) success rate after double-face BMG urethroplasty, with a median follow-up of 31 months. We hypothesized that the interposition of well-vascularized fatty tissue, analogous to the martius flap to support the ventral graft in the double-face BMG urethroplasty, could be the reason for this good surgical outcome. Finally, a patency rate of 100% in two patients was seen after staged augmented urethroplasty, as a result of the increased healing time after the first stage and therefore the possibility of tubularization on a well-vascularized graft bed in the second stage, at least 3 months later [16]. However, the long-term survival rates of grafts in this population still need to be studied, especially given the observation in cisgender men where grafts tend to result in lower success rates after long-term follow-up [22].

For strictures at the anastomosis between the fixed and native urethra, we only have data from two studies with small sample sizes. A 100% (1/1) recurrence was seen after HMS [16] and 44% (4/9) had a stricture relapse after ventral onlay BMG urethroplasty [17]. In this last study, no supportive tissue was used to optimize the vascularization of the BMG, which could have had an impact on graft survival rates. 

Lumen et al. reported a 25% recurrence rate after meatotomy (8 cases), 42% after HMS (19 cases). About half of the cases remain patent after both a free graft or pedicled flap urethroplasty. However, a patency rate of 70% was reported after a staged urethroplasty repair. Unfortunately, we cannot draw any conclusions based on these results, as the indication for each technique remains unclear. [12]

Only four of eight included studies described complications at three months after stricture treatment [15,16,17,18]. Verla et al. [15] were the only ones to describe their postoperative complications in detail, with only one patient having a CD grade III complication due to the placement of a suprapubic catheter because of acute urine retention. The most common complication after AR was fistula formation in 11% (5/44) of the cases. In contrast, this complication is absent after AR in cisgender men [22], presumably due to the excellent coverage of a bulbar urethroplasty with bulbospongious muscle and subcutaneous fat layers. Lumen et al. [16] reported that overall, none of the patients experienced any complications that were rated CD grade III after their various techniques for post-metoidioplasty strictures. However, no further details were described about the CD grade I and II complications. Therefore, no conclusions on complication rate can be drawn from this study. A possible explanation for the mild rhabdomyolysis (CD II) in a single case after graft urethroplasties [17] is the long operation time. However, this is only a hypothesis as no further details about the surgery time were described. 

Finally, Dabernig et al. [18] reported having no postoperative complications at all after their full free flap reconstructions for pan-urethral strictures. The complete absence of any postoperative complication after reconstruction with an RFFF seems very exceptional, due to the complexity of this surgery [18].

In cisgender patients, the dartos layer and the bulbospongious muscle are more developed compared to transgender men who thus lack this extra protective layer that could potentially provide bulk and vascular support to stricture repairs, in the region of the anastomosis between the fixed and pendulous urethra. In addition, the vascularization, as already mentioned above, is compromised and a very thin layer to cover these strictures gives a potentially higher risk of developing fistulas. Verla et al. [23] reported a similar rise in fistulization rate after stricture repair in failed hypospadias cases. Due to the ill developed dartos layer and often numerous previous procedures, only a thin layered coverage can be performed after stricture repair in these cases. 

Finally, only two studies reported on a functional outcome after urethral stricture repair, but neither provided a pre-operative assessment. [13,18] As a result, no comparison can be made with the patients’ preoperative functional status, and no valuable information can be given on this subject. 

### 4.2. Risk of Bias

All studies were found to be at high risk of bias using the 5-criterion quality appraisal checklist [10]. The quality of evidence on the management of urethral strictures after GGAS at present is low, highlighting the need for future research. 

### 4.3. Limitations

There are several limitations to our analysis. First, a variety in study populations were reported in the various included studies. As GGAS is performed both with phalloplasty and metoidioplasty using different flap and graft techniques, we could argue that these anatomical differences have effects on the characteristics of later developing strictures. Secondly, significant heterogeneity exists in regard to the various techniques in urethral stricture treatment (single stage, staged, local flaps, grafts, etc.). Additionally, all included studies were retrospective case series, which had a high risk of bias and reported on small sample sizes. This means that results may not be applicable to all individual cases, and no statistical methods could be conducted to assess any significant differences between techniques or groups of patients. Overall, only short-term follow-up after stricture repair, without any focus on patient reported outcome was reported. To date, no comparative data on stricture management in transmasculine patients exist. 

Nevertheless, this systematic review comprehensively summarizes the currently available evidence on this topic and identifies the knowledge gaps. The small cohorts and high risk of bias demonstrate the need for further investigation, both for surgical and functional outcome parameters. Prospective and comparative studies with larger sample sizes and homogeneous populations are highly needed, to develop robust clinical guidelines on stricture treatment as in cisgender patients. 

## 5. Conclusions

Different techniques have been described for the different clinical scenarios of urethral stricture disease. In the absence of comparative studies, however, it is impossible to advocate for one technique over another. This calls for additional research, ideally well-designed prospective RCTs focusing on both surgical and functional outcome parameters.

## Figures and Tables

**Table 1:** Summary characteristics and risk of bias of included studies. FtM (Female to Male), NA (Not Applicable), NR (Not Reported), RFFF (Radial Forearm Free Flap), ALT (Aterolateral Thigh flap), SCIAP (Superfical Circumflex Iliac Artery flap), IQR (Inter Quartile Range).

Year	Author	Type of Study	Follow-Up Nature	Funding	Study Participants	Date of Recuitment	Type of Gender Affirming Surgery	If Phalloplasty, Type of Urethral Lengthening	If Metoidioplasty, Type of Urethral Lenghthening	Mean/Median Follow-Up
2020	Lumen [16]	Case series	Retrospective	None	12/13 (92%) 1 stricture left conservative, 12/12 (100%) FtM	January 2006–March 2020	12/12 (100%) Metoidioplasty	NA	NR	15 (IQR: 10–42)
2020	Verla [15]	Case series	Prospective	None	44/44 (100%) FtM	January 2002–October 2019	44/44 (100%) Phalloplasty	33/44 (75%) RFFF tube in tube, 5/44 (11%) ALT tube in tube, 5/44 (11%) Pedicled SCIAP flap, 1/44 (2.3%) Other (not specified)	NA	40 (IQR: 7–125)
2020	Schardein [13]	Case series	Retrospective	None	9/9 (100%) FtM	December 2014–December 2019	9/9 (100%) Phalloplasty	9/9 (100%) RFFF tube in tube	NA	31 (range: 10–56)
2016	Wilson [14]	Case series	Retrospective	None	Mixed group(2/3–66.6% FtM) (1/3–33.3% oncologic penectomy)	May 2011–August 2015	2/2 (100%) Phalloplasty	2/2 (100%) RFFF tube in tube + Partially prelaminated with buccal mucosa	NA	8.7 (range: 6–13)
2015	Pariser [17]	Case series	Retrospective	None	Mixed group (9/10–90% FtM, 1/10–10% traumatic penile loss)	March 1998–June 2013	8/9 (88.9%) Phalloplasty, 1/9 (11.1%) Metoidioplasty	8/8 (100%) RFFF tube in tube	NR	9.5(range: 2.7-84)
2011	Lumen [12]	Case series	Retrospective	None	Mixed group (76/79–96.2% FtM, 3/79–3.8% penile insufficiency)	Aril 1994–May 2010	76/76 (100%) Phalloplasty	73/79 (92.4%) RFFF tube in tube, 6/79 (7.6%) UNCLEAR	NA	39 (range: 2–195)
2009	Lumen [11]	Case series	Retrospective	None	Mixed group (21/22–95.4% FtM, 1/22–4.5% traumatic penile loss)	September 2000–December 2008	21/21 (100%) Phalloplasty	20/22 (90.9%) RFFF tube in tube, 2/22 (9.1%) ALT tube in tube	NA	51 (range: 8–95)
2006	Dabernig [18]	Case series	Retrospective	None	Mixed group (6/9–66,7% FtM, 3/9–33.3% oncologic penectomy)	1999–2004	6/6 (100%) Phalloplasty	3/6 (50%) SCIP, 3/6 (50%) Abdominal	NA	41.8 (range: 13–55)

**Table 2:** Intervention and outcomes of included studies. DVIU (Direct Vision Internal Urethrotomy), HM (Heineke Miculicz), BMG (Buccal Mucosal Graft). CD (Clavien-Dindo), EPA (Exision and Primary Anastomosis) FtM (Female to Male), NA (Not Applicable), NR (Not Reported), IQR (Inter Quartile Range).

Author and Year	Mean/Median Age at Urethral Procedure (Months)	Stricture Time to Onset (Months)	Stricture Localization	Previous Endoscopic Procedures	Previous Meatotomy/Meatoplasty	Previous Urethroplasty	Urethrotomy (Otis/DVIU/Meatotomy/HM Stricturoplasty)	Augmented Urethroplasty with Graft	Augmented Urethroplasty with Local Flap	Primary Anastomotic Repair	Staged Urethroplasty with or without Augmentation	Definitive Preineal/Scrotal Urethrostomy	Perioperative Complications (Clavien Dindo)	Stricture Recurrence	Postoperative Complications
**Lumen et al. 2020 [16]**	30 (IQR:24–40)	9 (IQR: 12–17)	1/12 (8.3%) Anastomosis Native–Pars fixa, 4/12 (33.3%) Anastomosis Pars fixa–Pars pendulans, 4/12 (33.3%) Pars pendulans, 3/12 (24.9%) Meatal, 1/12 (8.3%) Panurethral	None	None	None	2/12 (17%) Meatotomy, 3/12 (25%) HM	None	1/12 (8.3%) Labium Minus flap (pan-urethral stricture)	None	6/12 (50%)	None	No CD ≥3, Lower grades not reported	1/3 (33.3%) after HM, 1/2 (50%) after meatotomy, 2/6 (33.3%) after staged repair, 0/1 (0%) after local flap repair	No Clavien Dindo complications ≥ 3, Lower NR
**Verla et al. 2020 [15]**	31 (IQR: 23–40)	10 (IQR: 6–22)	44/44 (100%) Anastomosis Pars fixa–Pars pendulans	11/44 (25%)	None	17/44 (39%)	None	None	None	44/44 (100%) EPA	None	None	11% CD 1, 14% CD2, 2.3% CD3 (Placement of suprapubic catheter)	19/44 (43%) After EPA repair	11% of patients CD I, 14% of patients CD II, 2.3% of patients CD III (placement of SPC) (3/44 (6.8%) UTI, 3/44 (6.8%) Wound infection, 2/44 (4.5%) Hematoma, 4/44 (9.1%) Retention, 5/44 (11%) Fistula)
**Schardein et al. 2020 [13]**	37 (range: 28–59)	NR	9/9 (100%) Anastomosis Pars fixa–Pars pendulans	NR	NR	NR	None	9/9 (100%) Double faced BMG	None	None	None	None	NR	2/8 (25%) after BMG repair, 1 case no information on follow-up	NR
**Wilson 2016 [14]**	32 Yo, 47 Yo	NR	2/4 (50%) Anastomosis Pars fixa–Pars pendulans, 1/4 (25%) Pars pendulans, 1/4 (25%) Meatal	NR	None	None	1/4 (25%) HM, 1/4 (25%) intervention not reported	2/4 (50%) BMG, both reinforced with fasciocuteaneous flap	None	None	None	None	NR	0/2 (0%) after BMG with flap, 0/1 (0%) after HM, 1 case no information on intervention or outcome	NR
**Pariser 2015 [17]**	39 (range: 26–56) Including cis gender patiënt	NR	9/9 (100%) Anastomosis Native–Pars fixa	9/9 100%	None	2/9 (22.2%)	None	1/9 (11.1%) Excision with dorsal anastomosis with ventral onlay BMG; 8/9 (88.9%) Incision with ventral onlay BMG	None	None	None	None	1/9 of patients CD1 (11.1%)	4/9 (44.4%) after BMG	1/9 of patients CD1 (11.1%) Mild rhabdomyolysis
**Lumen 2011 [12]**	37.6 (range: 19–65) Including cis gender patients	Overall median 23.5 (range: 13.5-31.2) 24.4 (meatal), 35.3 (pars pendulans), 13.5 (anastomosis pars pendulans - pars fixa), 28.1 (pars fixa)	18/118 (15.3%) Meatal, 28/118 (23.7%) Pars pendulans, 48/118 (40.7%) Anastomosis Pars fixa–Pars pendulans, 15/118 (12.7%) Pars fixa, 9/118 (7.6%) Multifocal	NR	NR	NR	8/118 (6.8%) Meatotomy, 19/118 (16.1%) HM	2/118 (1.7%) Free graft (type not reported)	10/118 (8.5%) Pedicled flap urethroplasty (3/10 were neo-scrotal pedicled flaps, 7/10 were neophallic skin flaps)	14/118 (11.9%) EPA	33/118 (28.0%) Johanson staged urethroplasty, 21/118 (17.8%) Perineostomy followed by urethral reconstruction, 10/118 (8.5%) Still at first stage of stage urethroplasty or perineal urethrostomy and awaiting further treatment	1/118 (0.8%)	NR	2/8 (25%) after meatotomy, 8/19 (42.1%) after HM, 6/14 (42.9%) after EPA, 1/2 (50%) after free graft, 4/10 (40%) after pedicled flap, 10/33 (30.3%) after staged repair, 13/21 (61.9%) after perineostomy with urethral reconstruction	NR
**Lumen 2009 [11]**	33 (range: 20–52) Including cis gender patients	20 (range: 1–90)	19/22 (86.4%) Anastomosis pars pendulans–Pars fixa 3/22 (13.6%) Pars pendulans	None	None	8/22 (36.4%)	32/32 (100%) DVIU (total of 32 procedures), (15/22 had 1 incision, 6/22 had 2 incisions, 1/22 had repetitive incisions) including cisgender patient	None	None	None	None	None	NR	12/22 (56.2%) after 1 DVIU	NR
**Dabernig 2006 [18]**	35.1 (range: 22–55) Including cis gender patients	NR	6/6 (100%) Pan-urethral	Yes, but percentage not reported	NR	Yes, but percentages not reported	None	None	6/6 (100%) Complete urethral reconstruction using RFFF	None	None	None	None	2/6 (33.3%) after complete free flap reconstruction	None

## Abbreviations

GGAS	Genital gender affirming surgery
DVIU	Direct vision internal urethrotomy
RFFF	Radial forearm free flap
ALT	Anterolateral thigh
SCIAP	Superficial circumflex iliac artery perforator
AF	Abdominal flap
QoL	Quality of Life
PROM	Patient reported outcome measure
HMS	Heineke Miculicz stricturoplasty
BMG	Buccal mucosal graft
GAU	Graft augmented urethroplasty
FAU	Flap augmented urethroplasty
AR	Anastomotic repair
EPA	Excision and primary anastomosis
RCT	Randomized controlled trial
PU	Perineal urethrotomy
SU	Scrotal urethrotomy
